# All-Cause and Cause-Specific Burden of Asthma in a Transitioning City in China: Population Study

**DOI:** 10.2196/44845

**Published:** 2024-11-14

**Authors:** Xuelin Cheng, Xiaoling Wu, Wenjing Ye, Yichen Chen, Peihua Fu, Wenchang Jia, Wei Zhang, Xiaoyun Xu, Di Gong, Changhua Mou, Wen Gu, Zheng Luo, Sunfang Jiang, Xiaopan Li

**Affiliations:** 1 Department of Health Management Center, Zhongshan Hospital, Shanghai Medical College of Fudan University, 180 Fenglin Rd, Xuhui, Shanghai, 200030, China, 86 13621925210; 2 Department of Respiratory Medicine, Xinhua Hospital, School of Medicine, Shanghai Jiao Tong University, Shanghai, China; 3 Office of Scientific Research and Information Management, Centre for Disease Control and Prevention, Pudong New Area, Shanghai, China; 4 Shanghai University of Medicine & Health Sciences Affiliated Zhoupu Hospital, Pudong New Area, 1500 Zhouyuan Rd., Pudong New Area,Shanghai University of Medicine & Health Sciences Affiliated Zhoupu Hospital, Shanghai, 201318, China; 5 Shanghai Children’s Medical Center, Shanghai Jiao Tong University School of Medicine, Shanghai, China; 6 Shanghai Engineering Research Center of AI Technology for Cardiopulmonary Diseases, Zhongshan Hospital, Fudan University, Shanghai, China

**Keywords:** asthma, mortality, years of life lost, trend analysis, decomposition method, Pudong

## Abstract

**Background:**

Understanding the impact of asthma on public health is crucial for evidence-based prevention and treatment strategies.

**Objective:**

This study aimed to identify the causes of asthma-related mortality in Pudong, Shanghai, China, offering insights for managing similar regions or countries in transition.

**Methods:**

Mortality statistics were obtained from the Vital Statistics System of Pudong for 2005‐2021. Temporal patterns for the burden of asthma were examined. The crude mortality rate (CMR), age-standardized mortality rate by Segi’s world standard population (ASMRW), and years of life lost (YLL) for both all-cause and asthma-specific deaths were computed. Mortality rates associating with aging and other variables were categorized using the decomposition technique. The autoregressive integrated moving average model was used to forecast the asthma-related death mortality rate by 2035.

**Results:**

A total of 1568 asthma-related deaths occurred during the follow-up period, with the CMR and ASMRW being 3.25/10^5^ and 1.22/10^5^ person-years, respectively. The primary underlying causes of death were chronic lower respiratory diseases, coronary heart diseases, and cerebrovascular disease. The YLL due to total asthma-related deaths added up to 14,837.76 years, with a YLL rate of 30.73/10^5^ person-years. Male individuals had more YLL (8941.81 vs 5895.95 y) and a higher YLL rate (37.12/10^5^ vs 24.38/10^5^ person-years) than female individuals. From 2005 to 2021, the ASMRW declined by 3.48%, and both the CMR and YLL rate decreased in the 0‐29, 70‐79, and ≥80 years age groups (all *P*<.01). However, asthma-related deaths increased from 329 people between 2005 and 2008 to 472 people between 2017 and 2021. The proportion of the population aged 80 years and older gradually increased by 1.43% (95% CI 0.20%-2.68%; *P*=.03), and the mortality rates of asthma deaths attributable to population aging rose by 21.97% (95% CI, 11.58%-33.32%; *P*<.001) annually.

**Conclusions:**

Asthma remains a significant public health challenge in transitioning countries, requiring increased attention and resource allocation.

## Introduction

Asthma is a diverse ailment, typically characterized by persistent airway inflammation. It is distinguished by a history of respiratory symptoms such as wheezing, breathlessness, tightness in the chest, and coughing that fluctuate in frequency and severity, accompanied by varying limitations in the ability to exhale [1]. Asthma poses a significant public health concern, impacting individuals of all ages worldwide [2]. A recent extensive survey of asthma among a nationally representative sample of Chinese adults highlighted the substantial public health challenge posed by asthma in China. The overall prevalence of asthma in the sample was 4.20%, equating to 45.70 million Chinese adults aged 20 years and older [3].

Not only does asthma impose a significant human and economic burden but it can also be fatal. In the 2019 Global Burden of Disease Study, asthma afflicted an estimated 262 million people and resulted in 461,000 deaths across 204 countries and territories [4]. The majority of asthma-related deaths occur in lower- and middle-income countries due to inadequate diagnosis and treatment. According to data from the China Pulmonary Health Study Group, only 28.8% of individuals with asthma reported receiving a diagnosis from a physician, and only 56% reported receiving treatment with inhaled corticosteroids, which is the gold standard treatment of asthma [3].

China is a transitioning country, and the prevalence of asthma has progressively risen over the last 20 years in China, likely as a result of rapid shifts in lifestyle and ecological conditions alongside the aging demographic [5]. Factors contributing to the increasing incidence and mortality rates of asthma include an aging population, financial resources, health care accessibility, medical advancements, and living conditions [6], which all concern China.

Pudong, Shanghai, is a global hub that is important to health care advancement and has a comprehensive public health system. It has been a prime example of China’s rapid economic growth since 1990 and covers an area of 1210 km^2^ [7]. In 2021, the population of Pudong was 3.17 million, which accounts for over 20% of Shanghai’s total permanent residents [8]. The Pudong New Area Mortality Registration System covers medical institutions at all levels and includes data on asthma-related mortality [9].

In this population-based study, we aimed to analyze asthma mortality rates and years of life lost (YLL) rates according to age or sex and their trends in Pudong from 2005 to 2021, as well as investigate population aging and other factors that may improve the prevention and management strategies in asthma.

## Methods

### Data Source

Data on asthma-related mortality spanning from 2005 to 2021 were sourced from the Pudong Mortality Registration System. The Public Security Bureau and the Statistics Bureau of Pudong meticulously provided comprehensive population data, which are essential for accurate analysis. Following standardized guidelines for determining the causes of death by the Chinese Disease Surveillance Points system [9], we rigorously conducted periodic assessments, data cleaning, and aggregation processes to uphold the integrity and comprehensiveness of the registration system [10]. This systematic approach encompassed various demographic variables, including age, sex, and cause of death, facilitating nuanced analyses of asthma-related deaths.

Asthma was defined following the *International Classification of Diseases, 10th Revision* [11] codes J45 to J46. All deaths for asthma, including asthma as a direct or indirect cause, during follow-up registered in the Pudong Mortality Registration System were included in this study.

### Ethical Considerations

Our study did not involve intervention on participants, and the surveillance procedure was ethically sanctioned by the Pudong Center for Disease Control and Prevention’s ethics committee, ensuring adherence to strict ethical guidelines (IRB#2016-04-0586). Throughout our investigation, we upheld the principle of noninterference with participants and ensured the utmost confidentiality of data, safeguarding the privacy and rights of all individuals involved in the study.

### Statistical Analyses

Crude mortality rates (CMRs) and age-standardized mortality rates by Segi’s world standard population (ASMRWs) were expressed per 100,000 person-years. The Poisson approximation technique was utilized to compare the CMR between sexes, while the Mantel-Haenszel test was used to compare the ASMRW. In our endeavor to identify and prioritize causes of premature mortality, we computed YLL using a methodology outlined in the Global Burden of Disease Study [12]. The YLL formula utilized by the World Health Organization [13,14] is provided below:


$$\begin{array}{l} YLL=\frac{KCe^{ra}}{(r+\beta {)}^{2}}\left\{e^{-(r+\beta )(L+a)}\left[-(r+\beta )(L+a)-1\right]\;-e^{-(r+\beta )a}\left[-(r+\beta )a-1\right]\right\}+\frac{\left(1k\right)}{r}(1-e^{-rL}) \end{array}$$

where *C* is the age weighting fit with constant (*C*=0.1658); *r* is the discount rate (*r*=3%); *a* is age at death; *β* is the age weighting parameter (*β*=0.04); *L* is the average life expectancy at the time of death, as determined by the standard reference life table from the Global Burden of Disease study [15]; and *e* is the Napier constant.

Age was stratified into 0‐4, 5‐14, 15‐29, 30‐44, 45‐59, 60‐69, 70‐79, and ≥80 years. Due to the low number of asthma-related deaths among individuals aged <30 years, trends in age-specific CMRs, ASMRWs, and YLL rates were analyzed for the age groups 0‐29, 30‐44, 45‐59, 60‐69, 70‐79, and ≥80 years. Temporal trends in CMRs, ASMRWs, and YLL rates were assessed using the Joinpoint Regression Program (version 4.3.1.0; National Cancer Institute) and reported as average annual percent change (AAPC) with the corresponding 95% CI. The significance of the AAPC was evaluated using the *z* test to ascertain whether it deviated significantly from zero. Terms such as “increase” or “decrease” were employed to characterize AAPCs that exhibited statistical significance (*P*<.05), while “stable” was used to denote nonsignificant trends. The autoregressive integrated moving average model was used to forecast the ASMRW in 2035. The autoregressive integrated moving average model is an extension of the basic autoregressive moving average model and is particularly useful for analyzing and predicting data with a time component. All statistical analyses were conducted utilizing SPSS (version 21.0; IBM Corp) and R (version 4.3.1; R Foundation for Statistical Computing). A *P* value of <.05 was deemed indicative of statistical significance.

## Results

### Baseline

During the period spanning from 2005 to 2021, there were a total of 362,558 deaths in Pudong, of which 1568 were asthma related. Among these, 920 (58.67%) deaths among male individuals and 648 (41.33%) deaths among female individuals were attributed to asthma. The median age at the time of death from asthma was determined to be 80.24 years, with an average age of 75.83 (SD 15.27) years. The CMR and ASMRW for asthma-related deaths were 3.25/10^5^ and 1.22/10^5^ person-years, respectively. For male individuals, the CMR and ASMRW were 3.82/10^5^ and 1.65/10^5^ person-years, respectively, while for female individuals, they were 2.68/10^5^ and 0.84/10^5^ person-years, respectively.

From 2005 to 2021, the YLL associated with asthma added up to 14,837.76 years, with a YLL rate of 30.73/10^5^ person-years. The YLL and YLL rate were higher in male individuals (8,941.81 y and 37.12/10^5^ person-years, respectively) than in female individuals (3,480.88 y and 16.55/10^5^ person-years, respectively). The primary comorbidities among underlying causes of death were chronic lower respiratory diseases except for asthma (J40-J44 and J47), coronary heart diseases (I20-I25), and cerebrovascular disease (I60-I69), accounting for 12.12% (190/1568), 9.63% (151/1568), and 6.89% (108/1068), respectively (Table 1). The CMR, ASMRW, average age, and median age at death by sex and cause of death are outlined in Table 2.

**Table 1.** Underlying causes of all asthma-related deaths in male and female individuals from 2005 to 2021 in Pudong, Shanghai, China.

Sex and rank		Underlying cause of death	Deaths, n (%)
**Male individuals (n=920)**			
	1	Asthma (J45-J46)	502 (54.57)
	2	Chronic lower respiratory diseases, except asthma (J40-J44 and J47)	119 (12.93)
	3	Heart diseases (I05-I09, I11, and I20-I52)	89 (9.67)
	4	Cerebrovascular disease (I60-I69)	57 (6.2)
	5	Lung cancer (C33-C34)	37 (4.02)
	6	Diabetes mellitus (E10-E14)	12 (1.3)
	7	Colorectal cancer (C18-C21)	12 (1.3)
	8	Prostate cancer (C61)	8 (0.87)
	9	Stomach cancer (C16)	7 (0.76)
	10	Falls (W00-W19)	5 (0.54)
**Female individuals (n=648)**			
	1	Asthma (J45-J46)	356 (54.94)
	2	Heart diseases (I05-I09, I11, and I20-I52)	82 (12.65)
	3	Chronic lower respiratory diseases, except asthma (J40-J44 and J47)	71 (10.96)
	4	Cerebrovascular disease (I60-I69)	51 (7.87)
	5	Diabetes mellitus (E10-E14)	15 (2.31)
	6	Lung cancer (C33-C34)	12 (1.85)
	7	Sequelae of external causes of morbidity and mortality (Y85-Y89)	10 (1.54)
	8	Falls (W00-W19)	8 (1.23)
	9	Colorectal cancer (C18-C21)	5 (0.77)
	10	Other diseases of intestines (K55-K63)	4 (0.62)

**Table 2.** Baseline characteristics of deaths.

Characteristic	Deaths (n=1568), n (%)	Age (y), mean (SD)	Age (y), median	Age (y), range	CMR^a^ (/10^5^ person-years)	ASMRW^b^ (/10^5^ person-years)	YLL^c^ (y)	YLL rate(/10^5^ person-years)
**Sex**								
Male	920 (58.67)	73.92 (15.15)	77.87	4.73‐105.44	3.82	1.65	8941.81	37.12
Female	648 (41.33)	78.54 (15.05)	83.07	23.99‐104.37	2.68	0.84	5895.95	24.38
**Period**								
2005‐2008	329 (20.98)	72.83 (17.16)	78.98	4.73‐105.44	3.16	1.50	3451.14	33.13
2009‐2012	355 (22.64)	75.16 (15.26)	79.69	18.27‐102.21	3.22	1.29	3434.58	31.18
2013‐2016	412 (26.28)	75.77 (14.85)	80.78	23.10‐101.07	3.57	1.27	3919.36	34.01
2017‐2021	472 (30.1)	78.48 (13.79)	81.99	28.38‐104.37	3.08	0.94	4032.68	26.32
**The underlying causes of asthma-related deaths**								
Asthma (J45-J46)	858 (54.72)	73.73 (16.99)	79.21	4.73‐103.08	1.78	0.72	8896.02	18.43
Chronic lower respiratory diseases, except asthma (J40-J44 and J47)	190 (12.12)	79.28 (11.89)	81.53	35.09‐105.44	0.39	0.13	1523.61	3.16
Coronary heart diseases (I20-I25)	151 (9.63)	82.61 (9.44)	84.64	54.19‐99.55	0.31	0.10	1032.04	2.14
Cerebrovascular disease (I60-I69)	108 (6.89)	79.63 (11.02)	82.52	34.34‐96.45	0.22	0.07	845.94	1.75
Total asthma-related deaths	1568 (100)	75.83 (15.27)	80.24	4.73‐105.44	3.25	1.22	14837.76	30.73

a CMR: crude mortality rate.

b ASMRW: age-standardized mortality rate by Segi’s world standard population.

c YLL: years of life lost.

### The Burden of Asthma-Related Deaths According to Age Groups

The majority (1312/1568, 83.67%) of asthma-related deaths were in individuals aged >60 years. The mortality rate increases with age, experiencing a substantial rise from the age of 45 years and peaking at the age of 80 years and older (46.51/10^5^ person-years). In terms of YLL, the top 3 age groups were those aged ≥80, 45‐59, and 60‐69 years, amounting to 3953.46, 3586.32, and 2857.79 years, respectively. Meanwhile, the top 3 age groups in YLL rates were those aged ≥80, 70‐79, and 60‐69 years, with rates of 194.02/10^5^, 76.98/10^5^, and 42.12/10^5^ person-years, respectively (Table 3).

**Table 3.** Number of deaths, CMR^a^, YLL^b^, and YLL rate by age group.

Age group (y)	Deaths (n=1568), n (%)	CMR (/10^5^ person-years)	YLL (y)	YLL rate (/10^5^ person-years)
0‐4	1 (0.06)	0.063687	30.09999	1.659606
5‐9	1 (0.06)	0.037079	28.92934	0.898892
15‐29	16 (1.02)	0.232861	435.4909	5.775858
30‐44	49 (3.12)	0.520045	1187.042	10.83688
45‐59	189 (12.05)	1.704989	3586.324	29.05162
60‐69	202 (12.88)	3.579184	2857.79	42.11786
70‐79	307 (19.58)	10.27323	2758.626	76.98255
≥80	803 (51.21)	46.50791	3953.457	194.0223
Total	1568 (100)	3.732954	14837.76	30.73492

a CMR: crude mortality rate.

b YLL: years of life lost.

### Trends Analysis

From 2005 to 2021, there was a significant decrease in the rates of asthma-related deaths among individuals aged 0‐29 years (AAPC −28.28%, 95% CI −40.44% to −13.63%; *P*=.002) and 45‐59 years (AAPC −6.74%, 95% CI −10.53 % to −2.24%; *P*=.006), whereas the age groups 60‐69 years (AAPC 4.78%, 95% CI 2.69%-6.92%, *P*<.001) and ≥80 years (AAPC 1.43%, 95% CI 0.20%-2.68%; *P*=.03) witnessed an increase in the rates of asthma-related deaths.

From 2005 to 2021, there were discernible temporal patterns in the CMRs, ASMRWs, and YLL rates, as indicated by the modeled CMRs, ASMRWs, and YLL rates. The male ASMRW and overall ASMRW exhibited a reduction of −3.98% (95% CI −5.38% to −2.57%; *P*<.001) and −3.42% (95% CI −5.02% to −1.92%; *P*<.001), respectively, while the CMR and female ASMRW did not show any significant changes (CMR: *P*=.89 amd female ASMRW: *P*=.06).

**Figure 1.**
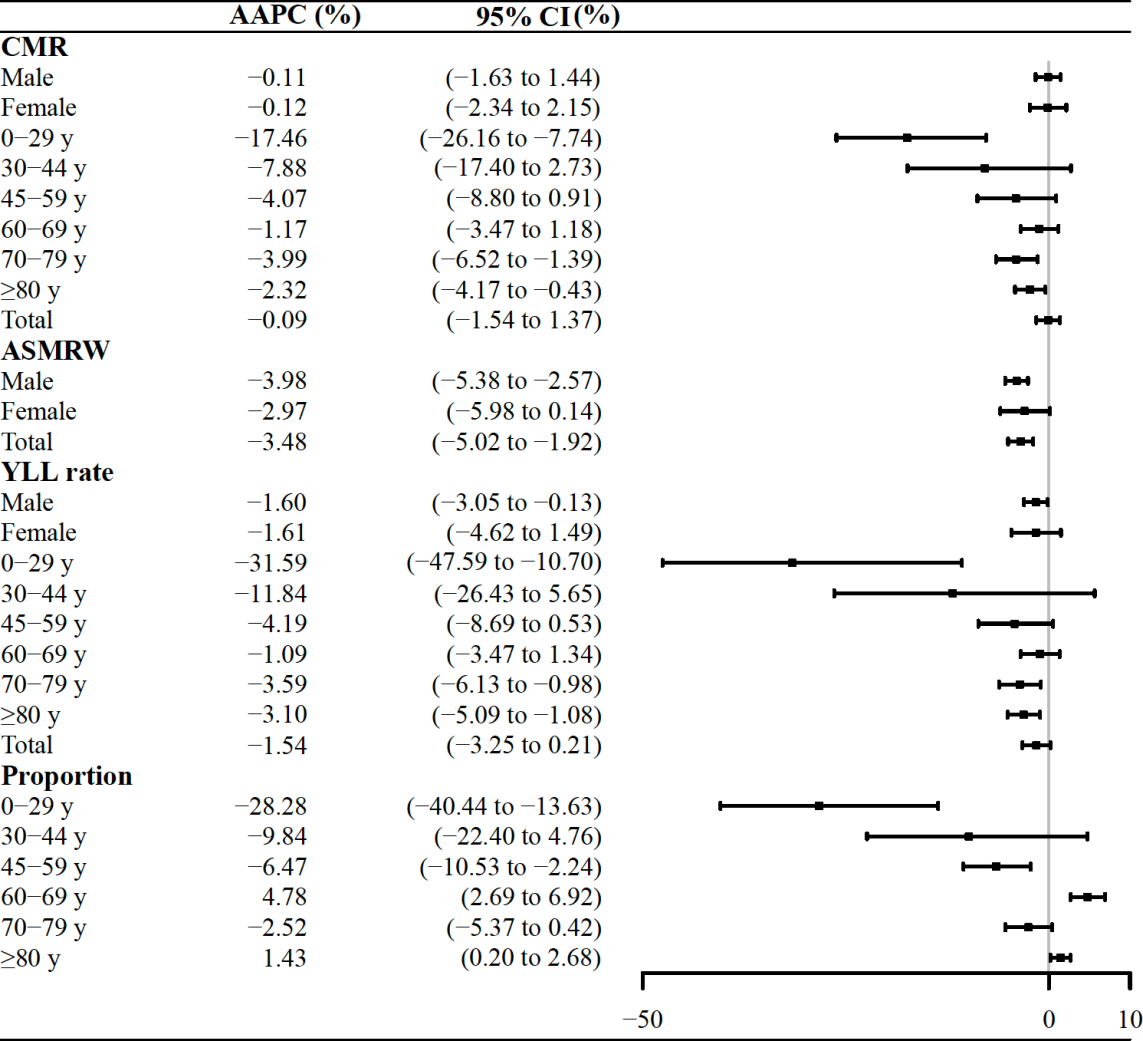
The trends in CMRs, ASMRWs, and YLL rates for asthma-related deaths in Pudong, Shanghai, China, from 2005 to 2021. AAPC: average annual percent change; ASMRW: age-standardized mortality rate by Segi’s world standard population; CMR: crude mortality rate; YLL: years of life lost.

**Figure 2.**
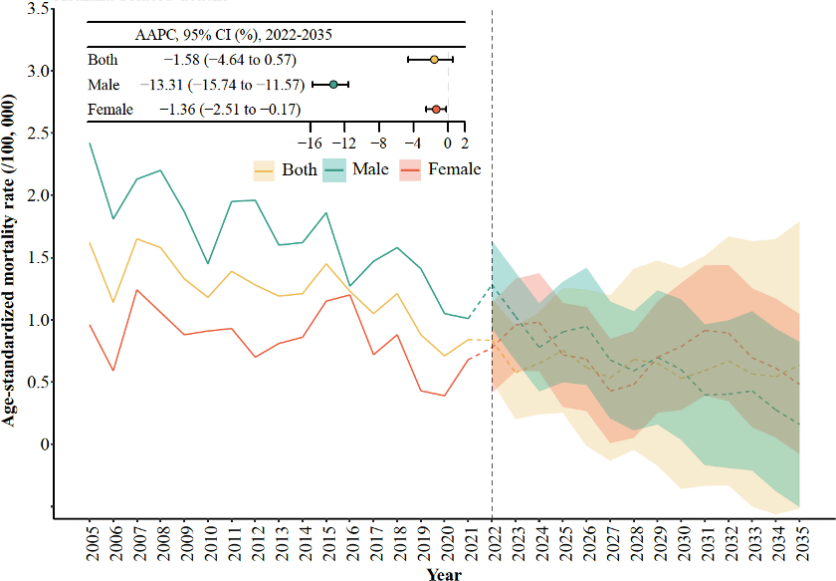
Forecasted age-standardized mortality rates for asthma-related deaths by 2035. AAPC: average annual percent change.

There was a notable decline in CMRs within the age groups of 0‐29, 70‐79, and ≥80 years. Additionally, there was a significant reduction in YLL rates within the same age groups of 0‐29, 70‐79, and ≥80 years. More details are shown in Figure 1. The projected ASMRW will continue to decline between 2022 and 2035. Compared to 2021, the ASMRW declined by 0.85/10^5^ person-years (AAPC −13.31%, 95% CI −15.74% to −11.57%) for male individuals and 0.20/10^5^ person-years (AAPC −1.36%, 95% CI −2.51% to −0.17%) for female individuals (Figure 2 and Multimedia Appendix 1).

### Quantitatively Impacts of Population Aging on CMR

**Figure 3.**
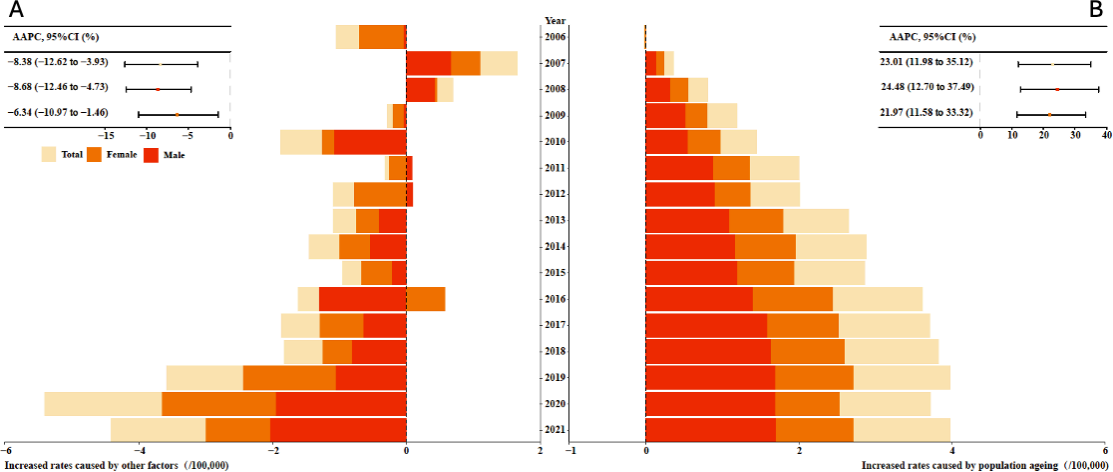
The increased mortality rates caused by (A) other factors and (B) population aging from 2005 to 2021 in Pudong, Shanghai, China, for both male and female individuals. AAPC: average annual percent change.

Analysis of the CMR data for asthma-related deaths in 2005 revealed a significant upward trend in mortality rates, particularly attributable to the aging population within the overall population, observed from 2006 to 2021, with an AAPC of 23.01% (95% CI 11.98%-35.12%; *P*<.001). Conversely, there was a marked downward trend in mortality rates attributed to other factors, with an AAPC of −8.38% (95% CI −12.62% to −3.93%; *P*=.001) during the same period. Among male individuals, the rate of mortality due to population aging increased by 24.48% (95% CI 12.70%-37.49%; *P*<.001), while the rate attributed to other factors decreased by −8.68% (95% CI −12.46% to −4.73%; *P*<.001). Among female individuals, the rate of mortality due to population aging rose by 21.97% (95% CI 11.58%-33.32%; *P*<.001), and the rate attributed to other factors decreased by −6.34% (95% CI −10.97% to −1.46%; *P*=.02). Further details can be found in Figure 3.

## Discussion

### Principal Findings

From 2005 to 2021, the CMRs, ASMRWs, and YLL rates for asthma-related deaths in Pudong showed decreasing trends, but the actual numbers continued to rise. The primary underlying causes of asthma-related deaths were chronic lower respiratory diseases (except asthma), coronary heart diseases, and cerebrovascular disease. Although the asthma-related mortality rate was declining, due to the unstandardized diagnosis and treatment of patients with asthma and the large population of China, asthma remains a serious health issue [3].

Both CMRs and YLL rates decreased across all age groups and sexes. Notably, asthma mortality was lower in the younger age group, a finding that was in line with several other studies. Other studies have also found that the mortality rate among people aged >20 years increases with age [16]. Compared to asthma mortality rates among children and adolescents worldwide [17], China indeed was at a lower level. This can be attributed to the effective management of asthma among a majority of young individuals, achieved through the use of standard treatments [18]. Additionally, the increased life expectancy has elevated the risk of developing asthma, resulting in a more significant number of asthma attacks and severe complications among older adult patients.

YLL take into account the age at which death occurs and the number of deaths at each age, providing a more comprehensive assessment of the impact of the disease beyond mortality statistics alone [19]. YLL could reflect the effect of asthma-related premature mortality on society, allowing for comparisons of disease burden and health status across different age groups. In this study, YLL were on the rise, even though YLL rates were declining, indicating that asthma remains a significant public health challenge that cannot be overlooked in transitioning regions, which was consistent with other research. Since 1990, the absolute number of chronic respiratory diseases has increased globally, but several age-standardized estimates have declined [20].

This study offers a comprehensive analysis of the impact of asthma-related deaths as a part of the overall mortality rate in a transitioning city in China [21]. The results indicate a decrease in mortality rates from asthma among individuals aged >70 years, which can be attributed to advancements in health care, public health initiatives, and environmental changes [22,23]. The mortality rates of cardiovascular diseases, diabetes, metastatic colorectal cancer, and other diseases in the Pudong also exhibited similar characteristics [21,23,24]. Additionally, the study highlights distinct patterns in asthma mortality across different age groups and underscores the influence of population aging and other factors on changing trends in asthma-related deaths. These findings underscore the importance of examining asthma’s incidence and impact to develop preventive measures to reduce the risk of severe illness or death and enhance overall health in China [25,26].

Our research also revealed that the primary concurrent illness in all instances of death arises from the circulatory and respiratory systems. There is also corroborating proof that various accompanying conditions, such as respiratory ailments and circulatory disorders, are predominant in all observed causes of death in patients with asthma [27]. A previous comprehensive analysis concluded that there was a link between asthma and an elevated likelihood of cardiovascular disease and overall mortality [28]. A further analysis of a large and ethnically diverse population revealed that individuals with persistent asthma had an increased incidence of cardiovascular disease events compared to those with occasional asthma or no asthma at all [29]. Older adults with asthma appear to face an increased risk of mortality, particularly when coupled with other illnesses, primarily chronic obstructive pulmonary disease [30,31]. Hence, treatment should be personalized based on the patient’s concurrent conditions [32,33].

The research suggested that mortality attributed to population aging has demonstrated a substantial increase, likely driven by the aging population and advancements in life expectancy. Aging can affect the severity and symptoms of asthma, as well as diagnosis and treatment, thereby increasing the complexity of managing asthma in older adults [34]. Other variables such as lifestyle, economic resources, health care services, medical advancements, and living conditions may help prevent premature deaths from asthma [30,31]. By 2050, it was projected that the global population aged 65 years and older would account for 17% of the total population, implying that the aging population with asthma would be living longer [35]. Addressing the relationship between population aging and asthma effectively is an urgent issue that needs to be resolved now.

A portion of the decline in asthma morbidity may be due to advancements in the comprehension of asthma regulation and treatment, as well as other potential factors. Despite the existence of relevant asthma treatment guidelines and standards in China, the current treatment situation in the country is not optimal due to the lack of medical resources in some areas and the low awareness among the public [36]. China needs to develop a national plan to enhance public awareness of asthma and promote standardized treatment, which is an important public health priority for reducing the burden of asthma [3]. Additionally, strengthening patient education is crucial for improving patient compliance. Patients should avoid allergen exposure, prevent respiratory infections, keep up with regular vaccinations, effectively manage comorbidities, and adhere strictly to their treatment plans [37].

Due to the scope of the analysis, this study has several significant limitations. First, our study was based on small, nonrepresentative samples from Pudong, necessitating a broader scope, including data from the entire country. Furthermore, the data available from death certificates for epidemiologic studies also present certain limitations, as they do not include information on the severity of asthma or exposure to risk factors such as occupational hazards or family history. Additionally, the study focused solely on mortality and did not address morbidity. Combining data on mortality and morbidity would enhance our understanding of the prevalence of asthma in China.

### Conclusion

Nowadays, Global Burden of Disease research is prevalent, and its data can be used to estimate the disease burden in different regions. Our study, grounded in real-world data, indicated a decline in the rate of premature asthma-related mortality, while the actual number of cases increased, thus providing a complementary perspective to Global Burden of Disease research. Therefore, asthma continues to pose a significant public health challenge in rapidly transitioning regions, underscoring the need for heightened attention in transitioning countries.

## Supplementary material

10.2196/44845 Multimedia Appendix 1 Age-standardized mortality rate by Segi’s world standard population according to sex.

## Abbreviations

AAPC: average annual percent change
ASMRW: age-standardized mortality rate by Segi’s world standard population
CMR: crude mortality rate
YLL: years of life lost
